# Supplementary material for: Geospatial Cellular Distribution of Cancer-Associated Fibroblasts Significantly Impacts Clinical Outcomes in Metastatic Clear Cell Renal Cell Carcinoma
Source: Cancers (Basel). 2021 Jul 26;13(15):3743. doi: 10.3390/cancers13153743 (PMC8345222; doi:10.3390/cancers13153743)
Supplement: Supplementary file 1 [file cancers-13-03743-s001.zip › cancers-1253010-supplementary.pdf]

cancers

MDPI

Supplementary Materials

Geospatial Cellular Distribution of Cancer-Associated Fibroblasts Significantly Impacts Clinical Outcomes in Metastatic Clear Cell Renal Cell Carcinoma

Nicholas H Chakiryan, Gregory J Kimmel, Youngchul Kim, Joseph O. Johnson, Noel Clark, Ali Hajiran, Andrew Chang, Ahmet M Aydin, Logan Zemp, Esther Katende, Jad Chahoud, Meghan C Ferrall-Fairbanks, Philippe E Spiess, Natasha Francis, Michelle Fournier, Jasreman Dhillon, Jong Y Park, Liang Wang, James J. Mulé, Philipp M Altrock and Brandon J Manley

A

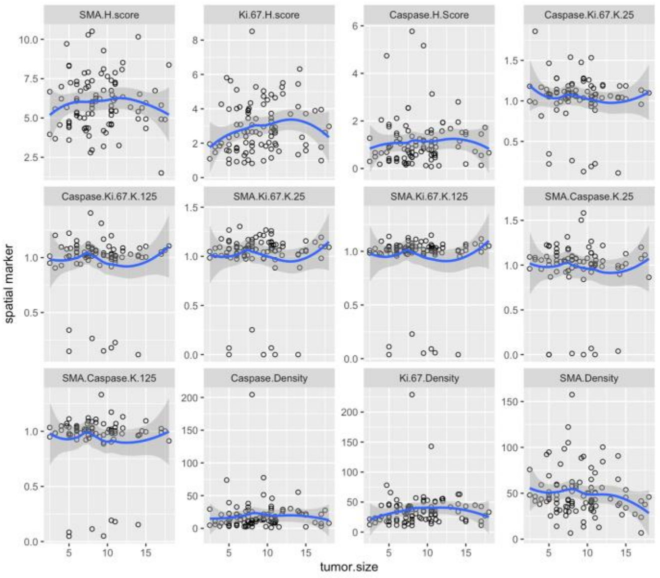

B

Variable	Correlation coefficient	95% CI		P
SMA.H.score	0.014	-0.187	0.214	0.894
Ki.67.H.score	0.115	-0.088	0.308	0.265
Caspase.H.Score	-0.018	-0.218	0.183	0.862
Caspase.Ki.67.K.25	-0.103	-0.31	0.114	0.352
Caspase.Ki.67.K.125	-0.019	-0.233	0.196	0.862
SMA.Ki.67.K.25	0.006	-0.197	0.209	0.952
SMA.Ki.67.K.125	0.031	-0.173	0.232	0.77
SMA.Caspase.K.25	-0.05	-0.264	0.169	0.655
SMA.Caspase.K.125	-0.036	-0.251	0.183	0.752
Caspase.Density	-0.023	-0.223	0.178	0.82
Ki.67.Density	0.01	-0.191	0.21	0.927
SMA.Density	-0.106	-0.3	0.096	0.304

Figure S1. A. Scatter plots and best-fit lines correlating tumor size with the immunohistochemistry-derived metrics used in the analysis. B. Correlation coefficients and their associated 95% confidence intervals and *p* values correlating tumor size with each immunohistochemistry-derived metric.
